# Influence of alveolar bone thickness and bucco-palatal inclination on root resorption of lateral incisors in unilateral maxillary impacted canines: a retrospective observational study

**DOI:** 10.1186/s12903-024-04076-1

**Published:** 2024-03-02

**Authors:** Weiman Sun, Yuanyuan Yang, Chenghuan Liu, Houxuan Li, Lang Lei

**Affiliations:** 1 Nanjing Stomatological Hospital, Affiliated Hospital of Medical School, Research Institute of Stomatology, Nanjing University, Nanjing, China; 2 Department of Periodontics, Nanjing Stomatological Hospital, Affiliated Hospital of Medical School, Research Institute of Stomatology, Nanjing University, Nanjing, China; 3 Department of Orthodontics, Nanjing Stomatological Hospital, Affiliated Hospital of Medical School, Research Institute of Stomatology, Nanjing University, #30 Zhongyang Road, Nanjing, 210018 China

**Keywords:** Maxillary impacted canine, Lateral incisor root resorption, Alveolar bone thickness, Incisor inclination, CBCT

## Abstract

**Background:**

This study aimed to investigate the potential associations between alveolar bone thickness, bucco-palatal inclination of maxillary lateral incisors, and lateral incisor root resorption in patients with unilateral maxillary impacted canines (UMICs).

**Methods:**

A total of three hundred and five subjects (120 males, 185 females; mean age, 16.39 years; standard deviation, 4.04) with UMICs were included. Canine position and root resorption were assessed using CBCT. UMICs were divided into palatal, buccal and mid-alveolus groups (PICs, BICs and MAICs), with 117, 137 and 51 subjects, respectively. Alveolar bone thickness and bucco-palatal inclination of lateral incisors were measured using the Dolphin software.

**Results:**

The prevalence of lateral incisor root resorption was significantly lower in the BICs (13.9%) than MAICs (29.4%) and PICs (29.1%). Alveolar bone thickness of the apical area was smaller in UMICs with lateral incisor root resorption than no resorption in both PICs (8.33 ± 1.64 vs 10.53 ± 2.55 mm) and BICs (8.94 ± 1.85 vs 10.76 ± 2.28 mm). Furthermore, lateral incisors on the impacted side were more buccally inclined in PICs with lateral incisor root resorption than no resorption, while in both BICs and MAICs, there was no statistical difference between lateral incisor root resorption than no resorption. Moreover, alveolar bone thickness of the apical area, rather than bucco-palatal inclination of lateral incisors, was significantly correlated with lateral incisor root resorption both in PICs and BICs.

**Conclusions:**

Lateral incisor root resorption is less common in BICs. Thinner alveolar bone thickness at the apical area of lateral incisors can be considered as a potential risk factor for lateral incisor root resorption in UMICs.

## Background

The impaction of canine is reported to be between 1 and 3% in orthodontic patients, and unilateral maxillary impacted canines (UMICs) account for a substantial part [1–3]. Root resorption of adjacent teeth, particularly the lateral incisors, is the most common complication associated with impacted canines, necessitating special attention during orthodontic treatment planning [4, 5]. Depending on various radiographic methods, the prevalence of root resorption associated with MICs ranges from 12.5% to 69.6% [6–8]. Maxillary lateral incisor is the most frequent resorbed neighboring teeth, accounting for up to 92% of all cases [8–10].

The etiology of root resorption in patients with impacted canine has not been fully uncovered. Several possible risk factors have been suggested, such as patient gender, canine apex, vertical canine crown position, canine magnification, the canine distance to the midline and so on [11–13]. It is noteworthy that almost all the instances of root resorption occur at the contact area between the impacted canine and adjacent teeth, indicating that close contact may be the cause of root resorption [9, 14]. Such physical proximity may build up the physiologic pressure around the roots of neighboring teeth [15, 16]. The contact was supposed to be avoided by aberrant lateral incisors, such as peg-shaped, based on their short roots [17].

The inclination of the lateral incisors was found to correlate positively with the proximity of canine crowns to the roots of lateral incisors. Close contact between the crown of palatal impacted canines and the root of the lateral incisors can lead to increased buccal inclination of the root of lateral incisors, suggesting that movement of the lateral incisor root may occur under pressure from the canine crown or its dental follicle [18].

The tooth buds and the roots are housed within the alveolar process; therefore, the anatomic features of the alveolar bone, especially the bone thickness, may affect eruption of dental follicles of the canines and the migration of the root of neighboring tooth. A statistically significant correlation was reported between alveolar bone thickness (ABT) and bucco-palatal inclination (BPI) of the upper incisor [19]. Thus, we hypothesized that a thick alveolar bone may allow for more space for bucco-lingual movement of lateral incisors, leading to less contact between the dental follicle of impacted canines and the root of lateral incisors. Such correlation among the ABT, BPI and root resorption of lateral incisors in MICs has never been explored.

The purposes of the present study were to explore the pattern of lateral incisors root resorption (LIRR) in subjects with UMICs by using the cone-beam computed tomography (CBCT), to assess the ABT and BPI of lateral incisors, to evaluate the difference in LIRR among the buccal, palatal, and mid-alveolar impacted canines, to detect the difference in ABT and BPI of lateral incisors between subjects with LIRR and no LIRR, and to explore whether ABT and BPI were risk factors for LIRR in UMICs.

## Methods

This retrospective study was approved by the Institutional Ethics Committee (IRB number: KY-2020NL-064). A total of 305 patients (120 males and 185 females; mean age, 16.39 ± 4.04 years) with UMICs (117palatal, 137 buccal and 51 mid-alveolus impaction) were included, from the Department of Orthodontics, Nanjing Stomatological Hospital, Nanjing University between April 2017 and April 2023. All selected cases had pre-treatment CBCT images and fulfilled the inclusion criteria.

The inclusion criteria for all patients are (1) Unilateral maxillary canine impaction; (2) Individuals aged from 12 to 25 years old; (3) Pre-treatment CBCT image data with clear and high quality. The exclusion criteria were (1) Bilateral canine impactions; (2) Pathology in the maxilla (cysts, odontoma, etc.); (3) At least one congenitally absent maxillary anterior tooth; (4) Maxillary anterior tooth with caries, severe periodontal diseases or after treatment of these diseases; (5) Pre-existing or in the stage of orthodontic treatment.

The sample size was determined using regression analysis. Following the methodology outlined by Green [20], minimum number of subjects in our study should be 66. We surpassed the minimum inclusion threshold.

All CBCT (NewTom VG, Italy) images were obtained (radiological parameters: 110 kV, 25 mA; voxel size, 0.25 mm; field of view, 16 cm × 16 cm; exposure time of 20–25 s for all subjects). Then, the images were converted to the CBCT-based Digital Imaging and Communications in Medicine (DICOM) format and evaluated via Dolphin Imaging Software (Chatsworth, CA, Version 11.95).

The following information was assessed and recorded. Canine crown position in relation to adjacent teeth was divided into palatal, buccal or mid-alveolus impacted canines (PICs, BICs and MAICs). Impacted canine crown mesiodistal position was divided into as Ericson and Kurol [21]: Sector 1 (S1): canine tip between midline and distal surface of lateral incisor; S2: canine tip between midline and distal surface of lateral incisor; S3: canine tip between midline of lateral incisor and distal surface of central incisor; S4: canine tip between distal surface and midline of central incisor; S5: canine tip between midline of central incisor and midline of the maxillary arch. Presence or absence of root resorption of adjacent root was assessed on 3D images. According to Ericson and Kurol [22], severity of root resorption was graded as: slight, moderate, severe. Vertical location of root resorption refers to Chaushu et al. [23]: cervical, middle and apical third.

**Fig. 1:**
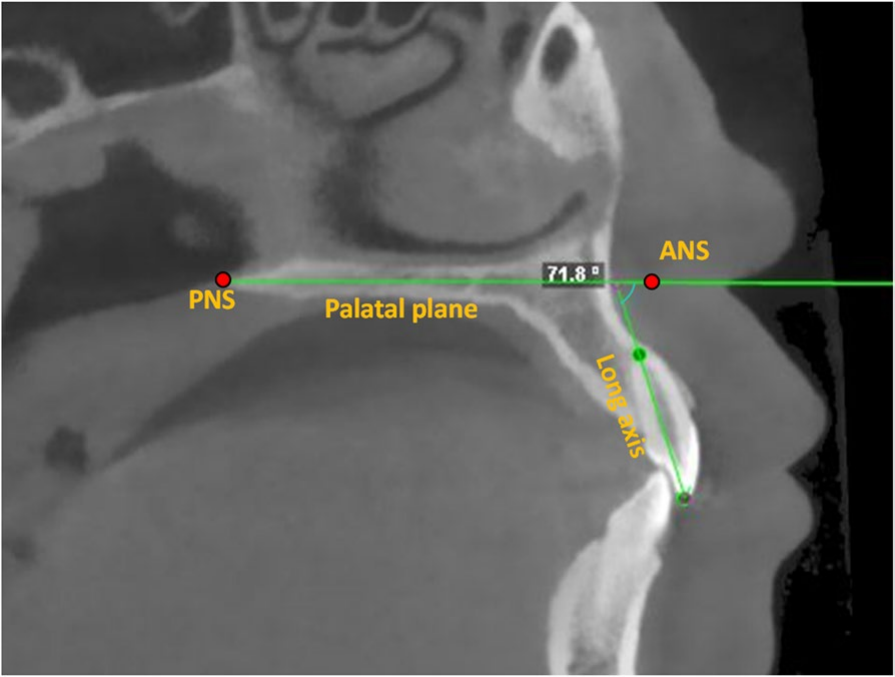
Measurement of bucco-palatal inclination of lateral incisors in CBCT. Bucco-palatal inclination of the maxillary lateral incisors were measured by the angle formed by the long axis of the maxillary lateral incisors and the palatal plane (ANS-PNS) in the sagittal plane. The larger the angle, the more labial inclination the root of the lateral incisor was

**Fig. 2:**
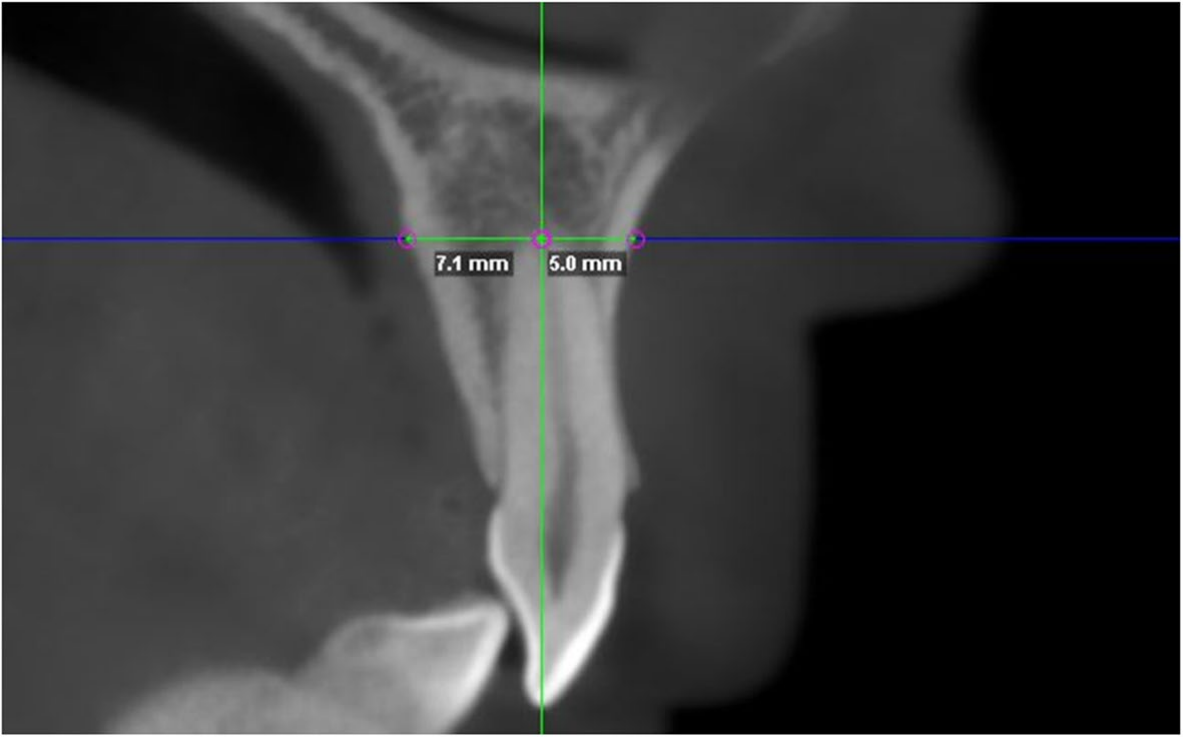
Measurement of the alveolar bone thickness at apical area of lateral incisors in CBCT. In the sagittal plane, alveolar bone thickness was evaluated in a bucco-lingual direction perpendicular to the long axis of each tooth at the root apex

Sagittal slices of each CBCT image were obtained. BPI of the maxillary lateral incisors were measured by the angle formed by the long axis of the maxillary lateral incisors and the palatal plane which connects the anterior nasal spine (ANS) and posterior nasal spine (PNS) in the maxilla [19] (Fig. 1). ABT was evaluated in a bucco-lingual direction perpendicular to the long axis of each tooth at the root apex [24] (Fig. 2).

### Statistical analysis

Statistical analyses were performed using the SPSS version 26 software (Chicago, IL, USA). A random sample of 31 CBCT images (10% of total sample) was re-evaluated after 1-month interval. The same examiner repeated all measurements to test intra-examiner reliability. Cohen kappa and the Intraclass Correlation Coefficient (ICC) were employed for assessing categorical and continuous variables, respectively.

Descriptive statistics were performed for all the measured variables. One-way ANOVA was conducted to explore the difference of ABT and BPI in groups of PICs, BICs and MAICs. An independent t-test was performed to compare differences between resorption and no resorption. In addition, categorical variables were tested by using chi-square test. Binary logistic regression analysis was used to determine the potential relationship among root resorption, ABT and BPI. The significance level was established at 0.05 level.

## Results

In our study, the values of Cohen kappa of categorical variables ranged from 0.91 to 0.93. The values of ICC for continuous variables were all greater than 0.85, indicating the high reliability of the data.

A total of 78 patients (25.6%) had at least one adjacent tooth resorption. Lateral incisors accounted for 87.2% of all the resorption cases, with root resorption in 20.5% of central incisors and 2.6% of first premolars in these subjects. Among the resorption cases of lateral incisors, 55.9% were considered slight, 25.0% moderate, and 19.1% severe, respectively. 73.5% of the resorption of lateral incisors were located at the apical third, 23.5% in the middle third and 2.9% in the cervical third (Table 1).

**Table 1:** Incidence, severity, and location of root resorption

	Number	Proportion(%)
Root resorption		
Yes	78	25.6%
No	227	74.4%
Location of root resorption		
Lateral incisor	68	87.2%
Lateral incisor + Central incisor	16	20.5%
Lateral incisor + First premolar	2	2.6%
Only central incisor	4	5.1%
Only first premolar	6	7.7%
Severity of root resorption of lateral incisor		
Slight	38	55.9%
Moderate	17	25.0%
Severe	13	19.1%
Location of root resorption of lateral incisor		
Apical third	50	73.5%
Middle third	16	23.5%
Cervical third	2	2.9%

All impacted canines with or without lateral incisor root resorption were compared as followed. No difference in the age, gender and affected side were detected between the subjects with LIRR and no-LIRR. However, bucco-lingual position of the maxillary canine significantly affected the occurrence of LIRR, with less LIRR in BICs (13.9%) than PICs (29.1%) and MAICs (29.4%). In addition, a significant distinction exists in the mesiodistal positioning of the maxillary canine. When the canine tip is located between the distal surface and the midline of the central incisor (S4), it exhibits the highest proportion (47.2%), as indicated in Table 2.

**Table 2:** Descriptive data regarding UMICs with LIRR and without LIRR

	LIRR	No-LIRR	X^2^	*P* value
Age (mean ± SD)	16.76 ± 4.07	16.29 ± 4.03	-	0.39
Gender			0.60	0.48
Female	44(23.8%)	141(76.2%)		
Male	24(20.0%)	96(80.0%)		
Affected side			0.30	0.68
Right	43(25%)	129(75%)		
Left	35(21.2%)	130(78.8%)		
Canine crown position			10.20	0.006**
Palatal	34(29.1%)	83(70.9%)		
Buccal	19(13.9%)	118(86.1%)		
Midalveolus	15(29.4%)	36(70.6%)		
Mesiodistal location			20.85	< 0.001***
S1	89(87.9%)	12(12.1%)		
S2	38(84.4%)	7(15.6%)		
S3	62(73.8%)	22(26.2%)		
S4	19(52.8%)	17(47.2%)		
S5	31(75.6%)	10(24.4%)		

Chi-square tests between LIRR and No-LIRR. *UMICs* indicates unilateral maxillary impacted canines, *LIRR* Lateral incisor root resorption, *SD* Standard deviation

^**^, *P* < .01

^***^, *P* < .001

Bucco-palatal inclination of the lateral incisors in UMICs was shown in Table 3. We first compared the difference among three groups: PICs, BICs and MAICs, and no difference in the inclination of lateral incisors was observed on the un-impacted side. While lateral incisors on the impacted side were significantly more palatally inclined in the PICs than the MAICs and BICs, with degree of 68.64 ± 13.55, 57.99 ± 12.52, and 56.60 ± 12.21, respectively. Furthermore, the difference between LIRR and no-LIRR was investigated within each of the three groups. It was found that significantly greater lingual inclination was observed in the no-LIRR than LIRR in PICs, 70.71 ± 13.48 degrees and 63.59 ± 12.53 degrees, respectively, while no difference was found in the BICs and MAICs.

**Table 3:** Bucco-palatal inclination (BPI) of the lateral incisors in UMICs ( Mean ± SD)

BPI	PICs	BICs	MAICs	PICs		BICs		MAICs	
				LIRR	No-LIRR	LIRR	No-LIRR	LIRR	No-LIRR
Impacted side	68.64 ± 13.55	56.60 ± 12.21^†††^	57.99 ± 12.52^†††^	63.59 ± 12.53**	70.71 ± 13.48	52.43 ± 11.52	57.28 ± 12.23	56.61 ± 7.54	58.56 ± 14.14
Un-impacted side	66.22 ± 9.08	64.82 ± 8.14	63.75 ± 7.82	65.44 ± 7.12	66.53 ± 9.77	63.03 ± 7.81	65.11 ± 8.19	63.14 ± 8.94	64.00 ± 7.43

*BPI* indicates bucco-palatal inclination, *UMICs* unilateral maxillary impacted canines, *SD* standard deviation, *LIRR* lateral incisor root resorption, *PICs* Palatal impacted canines, *BICs* Buccal impacted canines, *MAICs* Midalveolus impacted canines

^†††^, *p* < .001, vs PICs

^**^, *p* < .01, vs No-LIRR in three groups

The total alveolar bone thickness of the apical area (ABT-AA) in BICs (10.50 ± 2.30 mm) was thicker than PICs (9.89 ± 2.52 mm) and MAICs (9.60 ± 2.19 mm). Furthermore, we explored whether ABT-AA differed between LIRR and no-LIRR in BICs, PICs, and MAICs. ABT-AA was smaller in LIRR than no-LIRR in the PICs (8.33 ± 1.64 vs 10.53 ± 2.55 mm, *P* < 0.001) and BICs (8.94 ± 1.85 vs 10.76 ± 2.28 mm, *P* < 0.001), while no difference was found in MAICs. The similar results were seen in palatal ABT, while buccal alveolar thickness showed no statistical difference in all groups (Table 4).

**Table 4:** ABT at the apical area of lateral incisors on the un-impacted side( Mean ± SD)

ABT-AA	PICs	BICs	MAICs	PICs		BICs		MAICs	
				LIRR	No-LIRR	LIRR	No-LIRR	LIRR	No-LIRR
Labial	2.27 ± 1.06	3.21 ± 1.50	2.85 ± 1.56	2.11 ± 0.85	2.34 ± 1.13	3.11 ± 0.94	3.23 ± 1.57	3.28 ± 2.22	2.67 ± 1.19
Palatal	7.62 ± 2.53	7.29 ± 2.20	6.75 ± 1.83	6.22 ± 1.34***	8.19 ± 2.68	5.83 ± 1.41**	7.53 ± 2.22	6.94 ± 1.67	6.66 ± 1.91
Total	9.89 ± 2.52^†^	10.50 ± 2.30	9.60 ± 2.19^†^	8.33 ± 1.64***	10.53 ± 2.55	8.94 ± 1.85***	10.76 ± 2.28	10.22 ± 2.25	9.34 ± 2.14

*SD* indicates standard deviation, *ABT-AA* Alveolar bone thickness at the apical area, *PICs* Palatal impacted canines, *BICs* Buccal impacted canines, *MAICs* Midalveolus impacted canines, *LIRR* Lateral incisor root resorption

^†^, *p* < .05, vs BICs in total ABT-AA

^**^, *p* < .01, vs No-LIRR in the corresponding group

^***^, *p* < .001, vs No-LIRR in the corresponding group

The results of the binary logistic regressions can be viewed in Table 5. After finding the differences of UMICs with LIRR and no-LIRR in PICs and BICs, we further analyzed whether BPI and ABT were significant LIRR prediction factors. Apical ABT in group of PICs and BICs had negative correlation with the LIRR (OR: 0.599, *P* < 0.001; OR: 0.655, *P* = 0.003), indicating that each millimeter of ABT-AA reduction increased the likelihood of LIRR. However, BPI had no correlation with LIRR in two groups.

**Table 5:** Logistic regression analysis

	Coefficient	Standard error	Wald	*P* value	Odds ratio	Odds ratio 95% CI	
PICs							
ABT-AA	-0.513	0.132	15.029	< 0.001	0.599	0.62	0.776
BPI	-0.036	0.019	3.629	0.057	0.965	0.930	1.001
BICs							
ABT-AA	-0.277	0.142	8.892	0.003	0.655	0.496	0.865
BPI	-0.030	0.021	1.954	0.162	0.971	0.931	1.012

*PICs* indicates palatal impacted canines, *BICs* Buccal impacted canines, *CI* Confidence interval, *ABT-AA* Alveolar bone thickness at the apical area, *BPI* Bucco-palatal inclination

## Discussion

The mechanism of MICs and the occurrence of root resorption have not been fully understood. Palatal impaction of canines is thought to be caused by the absence of the correct guidance of lateral incisors or determined by genes, referred to as the guidance and the genetic theory, respectively [25–28]. In labial impacted canines, space deficiency may be an etiologic factor [29]. Therefore, in our study, both the buccal and palatal impacted canines were included and analyzed separately.

The prevalence of root resorption appears to vary depending on different population and detection methods. Root resorption in this study occurred in 25.6% of patients, and lateral incisor accounts for 87.2%. Consistent with most of results, the lateral incisor was the most affected in the adjacent teeth. Notably, regarding the resorption site, several sources in the literature have reported that the middle third of the maxillary incisor root is the most common position associated with MICs, followed by the apical third [30]. However, in our study, it was mainly located at the apical 1/3, aligning with the findings of Yan et al. [15]. Thus, in our study, we examined alveolar bone thickness at the apical area, revealing thinner dimensions in the resorption group compared to the non-resorption group.

Comparing the LIRR with no-LIRR group, there was no significant statistical difference in age, gender, and impacted side. However, the proportion of females was higher than males in the resorbed group in the western population, and root resorption was more prevalent in the dentition with a late dental development stage [11, 31, 32]. One interesting finding in the present study is that LIRR is less common in BICs than PICs and MAICs. Such difference has rarely been reported in the literature.

Contradictory results have been reported regarding the role of the dental follicle size, angulation, distance and vertical or horizontal relation of impacted canine in LIRR [11, 14]. In addition, crown-root proximity, is one potential cause of LIRR [3, 33]. However, this theory fails to explain the absence of LIRR in MICs with close crown-root proximity and the higher incidence of LIRR in BICs. Recently, Light et. al. reported different inclination of the lateral incisors between the impacted and un-impacted canines, suggesting that pressure from the crown of the impacted canine leads to the displacement of the adjacent teeth [18]. Our present study further demonstrated that lateral incisors with root resorption LIRR in PICs were more lingually inclined than no-LIRR, indicating that movement of the roots of lateral incisors may reduce the risk of LIRR in MICs. However, such difference was not observed in both BICs and MAICs. In contrast, lateral incisors in BICs with LIRR were more buccally proclined, indicating crown-root proximity and root displacement cannot fully explain the differed LIRR patterns in BICs and PICs.

Given that the dental buds of MICs and the root of lateral incisor are enclosed by the alveolus, we further explored whether ABT-AA may account for the LIRR in both BICs and PICs. A study has compared width of the alveolus (WA) at the level of the cervical margin of the adjacent teeth on the impacted and un-impacted side in the UMICs [34]. A clinical decrease in the WA on the affected side compared with the WA of the erupted canine; however, the LIRR was not explored. Since it is difficult in determining ABT on the impacted side and no statistical difference in the ABT was found between left and right side [35, 36], we measured the alveolar bone of lateral incisors on the un-impacted side, showing that thinner ABT-AA in LIRR than no-LIRR for both BICs and PICs. Therefore, our present study indicates that thinner ABT-AA might be a potential risk factor for LIRR.

To further substantiate our hypothesis, we conducted a logistic regression analysis incorporating the aforementioned positive findings. Because of the arithmetic relationship, only palatal ABT-AA was involved in this analysis. Due to no significant difference of BPI of un-impacted side, inclination of impaction side was included for analysis. The results of the binary logistic regressions suggested that ABT-AA rather than BPI can be thought as the risk factor of root resorption in PICs and BICs. It reminds us that ABT-AA should be considered for the prediction and diagnosis of MICs.

Nevertheless, our study still has several limitations. Root resorption is more frequent to be seen in UMICs, indicating the important role of local factors [3]. Since our study required contralateral lateral incisors as comparison, bilateral impacted canines were excluded. Besides, more subjects should be selected to investigate the risk factors of root resorption of other neighboring teeth, and the role of BPI and ABT-AA on the degree and location of root resorption should be further studied. In addition, it is inevitable that the possible potential of compensatory change of alveolar bone may be ignored, and further histologic and biologic studies should be performed to explain the potential mechanisms of all LIRR associated with MICs. Possible methods to predict root resorption, such as discriminant function equation based on many etiological factors as well as genetics [37, 38], may be required for clinician to adopt interceptive treatment approaches.

## Conclusions

Lateral incisor root resorption is less common in buccal impacted canines compared to palatal ones. The thickness of the alveolar bone at the apex of lateral incisors with root resorption in patients with unilateral maxillary impacted canines is thinner than those without resorption. The thickness of the alveolar bone at the apical area can be considered a potential risk factor for lateral incisor root resorption, whereas the bucco-palatal inclination of the lateral incisor was not.

## Data Availability

Data is provided within the manuscript or supplementary information files.

## Abbreviations

ABT: Alveolar bone thickness
BPI: Bucco-palatal inclination
LIRR: Lateral incisor root resorption
UMICs: Unilateral maxillary impacted canines
PICs: Palatal impacted canines
BICs: Buccal impacted canines
MAICs: Mid-alveolus impacted canines
ABT-AA: Alveolar bone thickness at the apical area
CBCT: Cone-beam computed tomography
ANS: Anterior nasal spine
PNS: Posterior nasal spine
ICC: Intraclass Correlation Coefficient
WA: Width of the alveolus
